# 

**Published:** 1899-03-11

**Authors:** 


					1 nuoriJA1Jt "'rue standard of highest purity."?Lancet, March Uth, 1899.
CADBURY'S mA TJf CADBURY'S
COCOA Jn| Wk COCOA
ABSOLUTELY PURE. NO DRUGS OR ALKALIES USED.
WHICH HOSPLTAJ.
No 650. Vol. XXV. [JgJSgJ*] Sattjeday>
March 11th, 1899.
[SuDHonptio^ernis.] pBICE TWOPENCE.

				

